# *Lactobacillus acidophilus* K301 Inhibits Atherogenesis via Induction of 24 (S), 25-Epoxycholesterol-Mediated ABCA1 and ABCG1 Production and Cholesterol Efflux in Macrophages

**DOI:** 10.1371/journal.pone.0154302

**Published:** 2016-04-27

**Authors:** Yi-Fan Hong, Hangeun Kim, Hye Sun Kim, Woo Jung Park, Joo-Yun Kim, Dae Kyun Chung

**Affiliations:** 1 Graduate School of Biotechnology and Institute of Life Science and Resources, Kyung Hee University, Yongin, 446–701, Republic of Korea; 2 Skin Biotechnology Center, Kyung Hee University, Yongin, 446–701, Republic of Korea; 3 RNA Inc., #308 Life Sceince Building, Kyung Hee University Global Campus, Yongin, 446–701, Republic of Korea; University of Cologne, GERMANY

## Abstract

*Lactobacillus acidophilus* species are well-known probiotics with the beneficial activity of regulating cholesterol levels. In this study, we showed that *L*. *acidophilus* K301 reduced the level of cholesterol through reverse transport in macrophages. *L*. *acidophilus* K301 upregulated the mRNA and protein levels of genes such as ATP-binding cassette A1 (ABCA1) and ATP-binding cassette G1 (ABCG1) under the control of liver X receptor (LXR), resulting in increased apoA-I-dependent cholesterol efflux in phorbol 12-myristate 13-acetate (PMA)-differentiated THP-1 cells. *L*. *acidophilus* K301 induced both ABCA1 and ABCG1 through the endogenous LXR agonist 24(*S*), 25-epoxcycholesterol, which is synthesized by intracellular cholesterol synthetic pathways. *In vivo* studies using *L*. *acidophilus* K301-treated *ApoE*^*-/-*^ mice showed reduced accumulation of lipoproteins in the arterial lumen. The inhibitory effects of *L*. *acidophilus* K301 on accumulation of lipoprotein in atherosclerotic plaques were mediated by the induction of squalene reductase (SQLE) and oxidosqualene cyclase (OSC) and resulted in ABCA1-mediated cholesterol efflux. Taken together, our findings revealed that *Lactobacillus acidophilus* K301 regulates the expression of genes related to cholesterol reverse transport via the induction of endogenous LXR agonist, suggesting the therapeutic potential of *Lactobacillus acidophilus* K301 as an anti-atherosclerotic agent.

## Introduction

Elevated serum cholesterol is a recognized risk factor associated with atherosclerosis and coronary heart disease [1–4]. Numerous drugs have been used to lower cholesterol levels in hypercholesterolemic individuals [5], although the undesirable side effects of these compounds have raised concerns about their therapeutic use [6]. The cholesterol accumulated on the inner wall of blood vessel can be removed by reverse cholesterol transport (RCT) from macrophages. RCT is mediated by LXR and ABC transporter system. Macrophages accumulated with high levels of cholesterol become foam cells and deposit on the blood vessel walls, which aggravates atherosclerotic lesion [7]. Cells control their cholesterol levels by three main mechanisms: regulation of synthesis, uptake (especially via low-density lipoprotein [LDL]), and efflux. Synthesis and uptake are largely governed by the sterol regulatory element binding protein (SREBP) family of transcription factors, whereas genes involved in cholesterol efflux are under the control of the oxysterol-activated liver X receptor (LXR) [8, 9].

The liver X receptors (LXRs) are members of the type 2 nuclear receptor family that are critical for the control of lipid homeostasis in vertebrates through binding to LXR response elements (LXREs) within the promoter regions of several responsive genes. These genes include ATP-binding cassette A1 (*ABCA1*) and ATP-binding cassette G1 (*ABCG1*), which are mainly involved in cholesterol reverse transport and mediate cellular cholesterol efflux in human and mouse macrophages to an extracellular acceptor and apolipoprotein E (ApoE). *In vivo* experiments using synthetic LXR agonists such as TO901317 have established that activation of LXR attenuates atherosclerosis [10]. Recent studies revealed that the LXR signaling pathways are important for the development of metabolic disorders such as hyperlipidemia and atherosclerosis [11]. Macrophage-specific deletion of LXRs in mice resulted in enhanced atherogenesis whereas liver-specific LXR overexpression decreased atherosclerosis [12]. Lipogenesis and triglyceride accumulation are enhanced by highly potent synthetic LXR agonists, which induce the expression of SREBP-1c, fatty acid synthetase (FAS), and lipoprotein ligase (LPL) [13].

Lactic acid bacteria (LAB) are components of the human gut microflora and are safe for use as probiotics. In particular, lipoteichoic acid, one of cell wall components of LAB, is known to regulate the immune system, including the anti-inflammatory response, and atherosclerotic plaque formation [14–16]. Ingestion of probiotic LAB has been reported to reduce serum cholesterol and LAB have been suggested as natural candidates for the prevention and treatment of hypercholesterolemia [17–19]. These hypocholesterolemic and anti-atherogenic effects have been explained by inhibition of absorption of dietary cholesterol by live LAB [20, 21]. However, to the best of our knowledge, there are no reports on the direct effect of LAB on LXR-related gene expression and cholesterol efflux. Therefore, the purpose of this study is to examine the effect of LAB on the induction of RCT from macrophages. This study investigated the influence of several *Lactobacilli* on expression of LXR-related genes, including *ABCA1* and *ABCG1*, in human PMA-differentiated THP-1 cells and mouse peritoneal macrophages. In addition, *Lactobacillus*-treated *ApoE* knockout (*ApoE*^*-/-*^) mice were used to examine factors that induce the progression of atherosclerosis such as serum cholesterol, infiltrated immune cells, and atherosclerotic markers.

## Materials and Methods

### Cell culture

THP-1 and HEK293 cells were obtained from the Korean Cell Line Bank (KCLB, Korea) and maintained in RPMI 1640 or MEM medium supplemented with 10% heat-inactivated fetal bovine serum (FBS), 100 U/ml penicillin, and 100 μg/ml streptomycin at 37°C in 5% CO_2_. Mouse peritoneal macrophage cells were maintained in RPMI 1640 (0.2% bovine serum albumin [BSA]), 100 U/ml penicillin, and 100 μg/ml streptomycin at 37°C in 5% CO_2_. THP-1 cells were plated in 12-well plates and differentiated to macrophages by the addition of 100 ng/ml phorbol 12-myristate 13-acetate (PMA) for 3 days. The differentiation of monocytes to macrophages was detected by expression changes in differentiation markers such as CD11b and chemokine receptor 2 (CCR2). Differentiated THP-1 cells were incubated for 12–16 h in FBS-free RPMI 1640 (0.2% BSA) with heat-killed lactic acid bacteria (LAB) or synthetic LXR agonist (TO901317).

### Preparation of lactic acid bacteria strains

The LAB strains used in this study were supplied by the R&D center of Maeil Dairy Industry Co (Gyeonggi, Korea). LAB strains were cultured with MRS broth (Difco Laboratories, MI, USA) at 30°C for 12–24 h and heat killed at 85°C for 15 min.

### Real-time PCR

Total RNA was isolated using Trizol reagent (Invitrogen, NY, USA) and DNase I and genomic DNA contamination was confirmed via optical density (O.D.) measurement and agarose gel electrophoresis. cDNA was synthesized from the isolated RNA using the Improm-II^™^ reverse transcription system (Promega, WI, USA). To quantify mRNA of LXR-regulated and LXR-related genes, real-time PCR amplification was carried out using the ABI prism 7000 sequence detection system (Applied BioSystems, CA, USA) and the PCR products were detected using SYBR Green. The primers used for real-time PCR are shown in S1 Table. The expression levels of the mRNAs were normalized to expression of glyceraldehyde-3-phosphate dehydrogenase (GAPDH) or β-actin.

### Immunofluorescence staining

TO901317 or *L*. *acidophilus* K301*-*stimulated THP-1 cells were incubated at 37°C for 1 h with 50 μg/ml human apoA-1 from human plasma in RPMI 1640. After incubation, THP-1 cells were fixed with 4% paraformaldehyde. Cells were blocked with phosphate-buffered saline (PBS) containing 1% BSA, and incubated with monoclonal anti-ABCA1 and polyclonal anti-apoA1 antibodies (Santa Cruz Biotechnology, CA, USA) in blocking buffer at room temperature. Donkey anti-mouse IgG-Alexa 488 and Donkey anti-goat IgG-Alexa 568 (Invitrogen) were added as secondary antibodies. Staining was examined by fluorescence microscopy.

### Determination of cholesterol efflux

Cholesterol efflux was measured using 1 μCi of [H^3^]-cholesterol/ml in the presence of heat- killed LAB strains or indicated doses of the LXR agonist TO901317. After 48 h of THP-1 monocyte differentiation into macrophages, the macrophages were labeled with [H^3^]-cholesterol in RPMI1640 containing 0.2% BSA for 24 h. The cells were washed twice with PBS and incubated for 6–12 h in RPMI1640 containing 0.2% BSA plus heat-killed LAB (1×10^8^/ml) or 1 μM TO901317. The macrophages were washed again with PBS and incubated in RPMI1640 containing 0.2% BSA in the presence and absence of apoA-I (10 μg/ml) for 8 h. The percentage cholesterol efflux was calculated by dividing medium-derived radioactivity by the sum of the radioactivity in the medium and cells [22].

### Transient DNA transfection and reporter assay

A luciferase reporter assay was used to determine the expression of LXR-related genes at the transcriptional level. Reporter constructs of the human ABCA1 promoter region were obtained by high-fidelity PCR with primers based on genomic sequences in Genbank (Accession No. AC012230). The promoter spans -919 nt to +239 nt and contains a binding site for the LXR receptor (LXRα/β) and the retinoid X receptor [18]. The product was ligated into pGL3-Basic (Promega) and designated pGL3-hABCA1. cDNA of human LXR-α was amplified from mRNA obtained from THP-1 cells by RT-PCR and cloned into pCMV-Tag 2A (Stratagene, CA, USA). The HEK 293 human embryonic kidney cell line was transfected with pCMV- LXR (0.25 μg) and pGL3-hABCA1 (0.5 μg). The pRL-SV40 vector (Promega) was also co-transfected for normalization of transfection efficiency.

### Western blot analysis

PMA differentiated THP-1 cells were cultured to confluency on 60-mm dishes in regular media. Cells were treated with compactin (0.1 to 10 μM) and/or *L*. *acidophilus* K301 in RPMI1640 containing 0.2% BSA for 24 h and then washed with PBS and harvested with lysis buffer (50 mM Tris (pH 7.5), 150 mM NaCl, 1 mM EDTA, 1% Triton X-100, 1% sodium deoxycholate, 0.1% SDS, 1 mM phenylmethylsulfonyl fluoride [PMSF], 5 g/ml aprotinin, 5 g/ml leupeptin). Quantification of proteins was performed by the Bradford assay (Sigma, MO, USA). SDS-PAGE was performed with a 4% stacking gel and a 8% (for ABCA1) or 10% (for ABCG1) resolving gel, followed by transfer to PVDF membranes (Bio-Rad, CA, USA). The membranes were blocked overnight at 4°C in blocking solution (5% skim milk in TBS-T) and then incubated with mouse monoclonal anti-ABCA1 antibody and rabbit polyclonal anti-ABCG1 antibody for 1 h at room temperature. Rabbit polyclonal anti-β-actin antibody was included as a housekeeping protein to normalize the total amount of protein. The membranes were washed with TBS-T and incubated with HRP-conjugated anti-mouse IgG or anti-rabbit IgG for 1 h at room temperature. The signal densities for specific bands on the western blots were quantified using Image Lab density analysis software (Version 2.2).

### Thin-layer chromatography

Thin-layer chromatography (TLC) was conducted for assessment of cholesterol and 24(S), 25-epoxcycholesterol (24, 25-EC) synthesis using a method detailed by Wong *et al*. [23]. Cells were incubated in 6-well plates in the presence or absence of inhibitors (compactin or RO488071) or *L*. *acidophilus* K301 together with [^14^C]-acetate (2 μCi/well) for 24 h. The medium was aspirated and the cells were washed twice with PBS. Cells were lysed in KOH in methanol (1.2 ml; 10% w/v) followed by the addition of 1.4 ml water. Butylated-hydroxy toluene/ethylenediamine tetraacetic acid (EDTA; 20 mM final concentration for each) was added to each sample, followed by saponification at 70°C for 1 h. After cooling, neutral lipids were extracted twice with n-hexane:diethyl ether (2 ml; 1:1 v/v). Extracts were dried, re-dissolved in n-hexane:diethyl ether (100 μl; 1:1 v/v), and separated by TLC with a mobile phase of n-hexane:diethyl ether:acetic acid (60:40:1 v/v/v). For visualization, TLC plates were exposed to X-ray film.

### Ethics statement

The mice were cared for and used in accordance with guidelines of the animal ethics committee of Kyung Hee University. Specific approval for the mouse experiments was obtained for protocol (KHU-14-021) by the Department of Laboratory Animal, Institutional Animal Care and Use Committee at Global campus of Kyung Hee University (Yongin, Korea). The research titled is “The proof of the anti-inflammatory effects of lactic acid bacterial cell wall components.” They were kept in individual cages at 24±2°C and 50±10% moisture condition, and fed nutritionally balanced rodent food (Central Lab. Animal Inc. Korea) and sterilized water. All reasonable efforts were made to ameliorate suffering, including use of anesthesia for painful procedures.

### *In vivo* studies

*ApoE* knockout (*ApoE*^*−/−*^) mice were purchased from the Jackson Laboratory (Bar Harbor, ME) and bred at the Samsung Biomedical Research Institute under specific pathogen-free conditions. Eight-week-old *ApoE*^*−/−*^ mice were randomly divided into two groups: a high-fat high-cholesterol (HFHC) diet and placebo (*n* = 6), or a HFHC diet and *L*. *acidophilus* K301 treatment (*n* = 6). The HFHC diet contained 0.15% cholesterol, 6% fat, 0.8% sodium chloride (CRF-1, Research Diets, Inc. NB, USA). To induce the atherogenic effects, the 8-week-old mice were fed with the HFHC diet for 8 weeks and then with normal chow for 8 weeks. During the last 8 weeks, animals were randomized for oral administration of *L*. *acidophilus* K301 (1×10^8^ CFU/kg) or an equal volume of placebo twice a week for 8 weeks. After completion of treatment with *L*. *acidophilus* K301 or placebo at the 16th week, the mice were sacrificed by CO_2_ inhalation. Total plasma cholesterol and triglyceride levels were measured using a 7020 Automatic Analyzer (Hitachi, Japan). To isolation and culture of peritoneal macrophages, euthanasia was performed with ketamine-xylazine given intraperitoneally. The peritoneal cavities were then flushed with 5 ml DMEM. The peritoneal lavage fluids were centrifuged at 1,500 rpm for 10 min and the cells were resuspended with ACK lysing buffer for 1 min. After washing 2 times, cells were resuspended with DMEM and plated. After incubation for 2 h at 37°C, the cells were washed and nonadherent cells were removed. Peritoneal macrophages were cultured at 37°C in DMEM added 10% FBS and penicillin/streptomycin.

### Histological examination

The cross-sectional areas of atherosclerotic lesions were quantified by evaluating the lesion size in the aortic sinus. Briefly, the heart and aorta were perfused with PBS for 10 min and with 4% paraformaldehyde for 5 min, and then fixed for at least 24 h. The isolated hearts and aortas were embedded in Tissue-Tek OCT compound (Sakura Finetechnical, Tokyo, Japan), and frozen at -70°C. All samples were sectioned using a cryostat at -20°C, and six consecutive 5-μm thick sections were cut from the aorta where the valve cusps were visible. Plaques were stained with Oil Red O and counterstained with hematoxylin. The lesion area (μm^2^) of three sections was quantified by computer-assisted morphometry (Image-Pro Plus, MD, USA), and the average lesion area was calculated for each animal. Immunohistochemical (IHC) studies were conducted using a commercially available kit (DAKO, USA) with anti-mouse macrophage/monocyte antibody (MOMA-2, Serotec, UK) and anti-mouse ABCA1 antibody (Santa Cruz Biotechnology). After the IHC reactions, the lesions that showed positive signal were quantified by calculating the lesion area (μm^2^) of three sections as described above.

### Statistical analysis

All experiments were performed at least three times. The data shown are representative results for the means ± standard deviation of triplicate experiments. Differences were judged to be statistically significant when the *P* value was less than 0.05.

## Results

### *L*. *acidophilus* strains increased the expression of ABCA1 and ABCG1

To examine the effects of LAB on *ABCA1* and *ABCG1* gene expression in macrophages, THP-1 cells were differentiated with PMA and the relative expression of monocyte-to-macrophage maturation markers was examined by real-time PCR. Increased gene expression of CD36 and CD11b was observed, whereas expression of chemokine receptor 2 (CCR2) was decreased during differentiation, indicating that monocyte-like THP-1 cells differentiated into macrophages. In addition, the cholesterol uptake ability was also increased in differentiated macrophages (S1 Fig).

**Fig 1:**
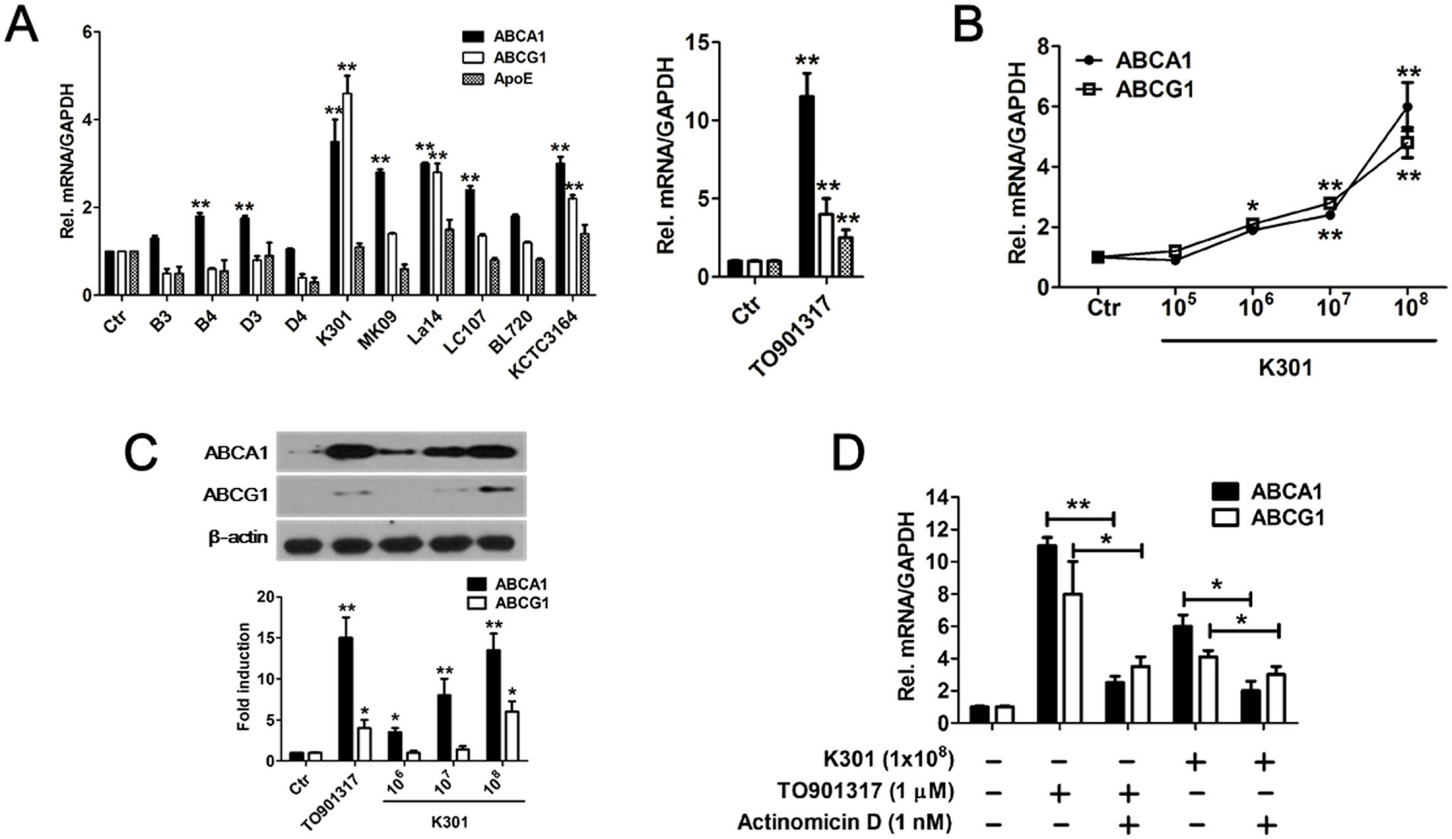
Effects of heat-killed lactic acid bacteria on LXR-related gene expression in macrophages. (A) THP-1 macrophages were incubated with 1×10^7^/ml heat-killed LAB or 1 μM TO901317. Expression of *ABCA1*, *ABCG1*, and *ApoE* mRNA was examined by real-time PCR. (B) Cells were incubated with the indicated dose of heat-killed *L*. *acidophilus* K301 and the expression of *ABCA1* and *ABCG1* was examined by real-time PCR. (C) The protein expression of ABCA1 and ABCG1 was examined by western blotting using specific anti-ABCA1 and anti-ABCG1 antibodies. The amount of protein expression was normalized to beta-actin, and fold changes were presented by densitometry analysis (lower panel). (D) Cells were treated with heat-killed *L*. *acidophilus* K301 or TO901317 in the presence or absence of 1 nM Actinomycin D. The expression of *ABCA1* and *ABCG1* was examined by real-time PCR. mRNA expression was normalized to expression of *GAPDH*. Data are given as meanta a. *P < 0.05; **P < 0.01 vs. control (Ctr). The error bars in individual samples are shown as variations from triplicate assays.

Ten different heat-killed lactic acid bacterial strains were examined for their ability to induce *ABCA1*, *ABCG1*, and *ApoE* gene expression in PMA-differentiated THP-1 macrophages through real-time PCR (S1 Table). *ABCA1* mRNA gene expression was significantly increased by *L*. *acidophilus* K301, *Bifidobacterium infantis* MK09, *L*. *acidophilus* La14, *Lactobacillus casei* LC107, and *L*. *acidophilus* KCTC 3164. In contrast, *L*. *paracasei* B3, *L*. *paracasei* B4, *L*. *plantarum* D3, *L*. *rhamnosus* D4, and *Bifidobacterium longum* BL720 induced low levels of *ABCA1* transcription (please see S2 Table for abbreviations of bacterial strains used in this study). Among the strains used in this study, *L*. *acidophilus* K301, *L*. *acidophilus* La14, and *L*. *acidophilu*s KCTC 3164 significantly increased *ABCG1* mRNA expression. While ABCA1 expressions were increased by LAB, some bacterial strains including *L*. *paracasei* B3, B4, and *L*. *rhamnosus* D4 inhibited ABCG1 and *ApoE* gene expression (Fig 1A). *L*. *acidophilus* K301, which was isolated from dairy products, had a dose-dependent effect on expression of *ABCA1* and *ABCG1* (Fig 1B). The protein level of ABCA1 and ABCG1 also increased in a dose-dependent manner when cells were treated with *L*. *acidophilus* K301 (Fig 1C, upper panel). Densitometry of western blots is shown in the lower panel of Fig 1C. To confirm the effects of *L*. *acidophilus* K301 on *ABCA1* and *ABCG1* mRNA expression, cells were treated with Actinomycin D prior to treatment with *L*. *acidophilus* K301. Actinomycin D prevents the increase of *ABCA1* and *ABCG1* mRNA expression as well as the increase in cholesterol efflux induced by the ligands [24]. Our results showed that *L*. *acidophilus* K301-mediated *ABCA1* and *ABCG1* mRNA expression was suppressed by Actinomycin D (Fig 1D). A synthetic LXR agonist, TO901317, was used as a positive control in this study. The effect of the LXR agonist TO901317 and *L*. *acidophilus* K301 on expression of lipogenesis-related gene expression was examined through microarray analysis (S2 Fig). Only *ABCA1* and *ABCG1* gene expression was increased, by 3-fold and 4-fold respectively. Expression of *LPL* decreased 3-fold compared to the control, indicating that *L*. *acidophilus* K301 may increase ABCA1-mediated cholesterol efflux [25]. The positive control TO901317 increased expression of both the *ABCA1* and *ABCG1* genes, in addition to *LPL*. These data suggest that *L*. *acidophilus* K301 can function as a LXR agonist.

### *L*. *acidophilus* K301-mediated ABCA1 and ABCG1 expression was associated with intracellular cholesterol synthesis

**Fig 2:**
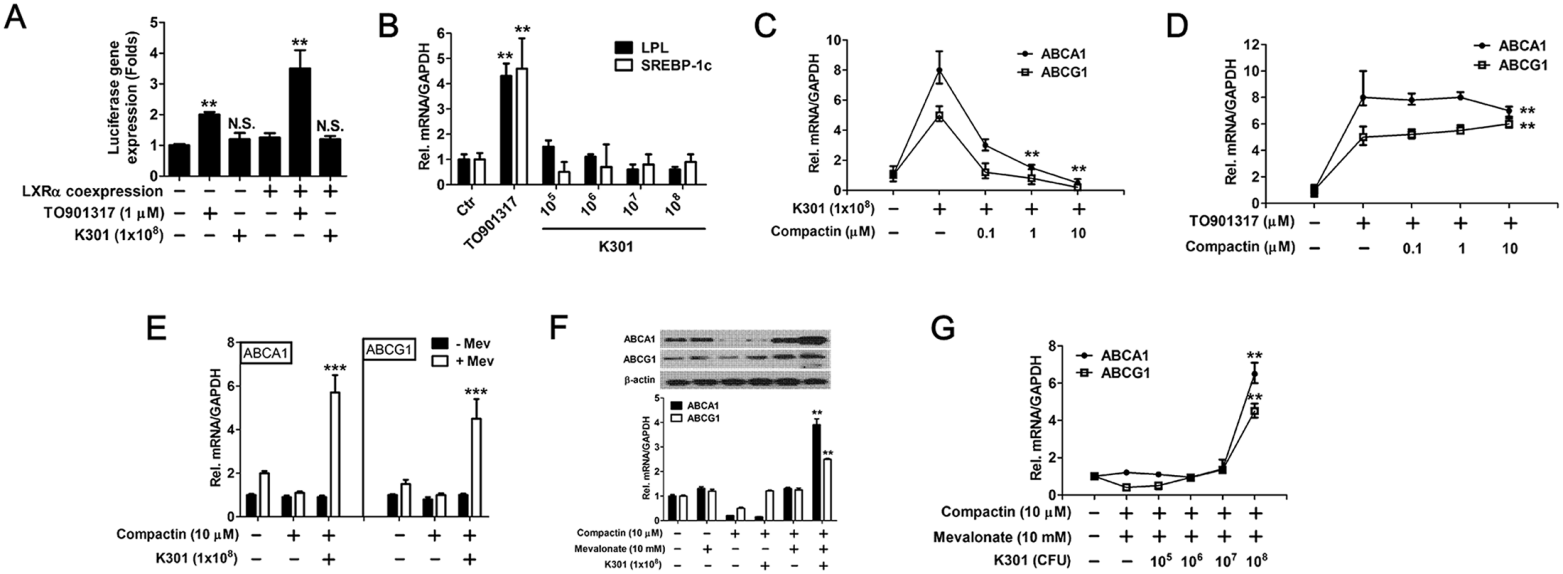
*L*. *acidophilus* K301-mediated ABCA1 and ABCG1 expression was associated with intracellular cholesterol synthesis. (A) Reporter assay of LXR agonistic activity of heat-killed LAB. A firefly luciferase reporter gene containing the human LXR binding site of the *ABCA1* promoter was constructed and transfected into HEK293 cells together with an expression vector containing human LXR-α. The reference plasmid pRL-SV40 was also co-transfected. Cells were stimulated with 1×10^8^ heat-killed *L*. *acidophilus* K301 or TO901317. Firefly luciferase activity was measured and normalized to *Renilla* luciferase activity. (B) PMA differentiated macrophages were stimulated with the indicated dose of heat-killed *L*. *acidophilus* K301 and mRNA expression of *LPL* and *SREBP-1c* was examined by real-time PCR. (C, D) Cells were treated with heat-killed *L*. *acidophilus* K301 (C) or with TO901317 (D) in the presence of compactin. mRNA expression of ABCA1 and ABCG1 was examined by real-time PCR. (E) Cells were stimulated with heat-killed *L*. *acidophilus* K301 in the presence of compactin and 10 mM mevalonate. *ABCA1* and *ABCG1* mRNA level was examined by real-time PCR. (F) Cells were stimulated with heat-killed *L*. *acidophilus* K301 in the presence of compactin and 10 mM mevalonate. The protein level of ABCA1 and ABCG1 was examined by western blotting, and corresponding densitometry data are shown (low panel). (G) Cells were stimulated with the indicated dose of heat-killed *L*. *acidophilus* K301 with 10 μM compactin and 10 mM mevalonate. The mRNA level of *ABCA1* and *ABCG1* was examined by real-time PCR. β-actin was used as a protein loading control; mRNA expression of genes was normalized to *GAPDH*. Results are the means of three independent experiments (mean±S.D). **P < 0.01; ***P < 0.001 vs. untreated cells. The error bars in individual samples are shown as variations from triplicate assays.

To investigate the effect of *L*. *acidophilus* K301 on LXR agonistic activity, pGL3-hABCA1 luciferase vector and pCMV- LXR expression vector were transfected into HEK293 cells. Luciferase assay was performed using cells treated with *L*. *acidophilus* K301 or the LXR agonist TO901317. The transcriptional activity of the hABCA1 promoter containing the LXR binding site was unchanged by *L*. *acidophilus* K301 in the pGL3-hABCA1 transfectants as well as in the pGL3-hABCA1 and pCMV-LXR co-transfectants, indicating that *L*. *acidophilus* K301 has no LXR agonist activity. In contrast, TO901317 significantly increased hABCA1 promoter activity in both transfectants (Fig 2A).

It is known that downregulation of lipoprotein lipase (LPL) increases ABCA1-mediated cholesterol efflux in THP-1 macrophages [25] and that sterol regulatory element binding protein-1 (SREBP-1) induces the expression of numerous genes involved in lipid metabolism, including LPL [26]. Although TO901317 significantly increased *LPL* and *SREBP-1c* mRNA expression, *L*. *acidophilus* K301 did not increase expression of these mRNAs in PMA-differentiated THP-1 macrophages, suggesting that *L*. *acidophilus* K301 may inhibit cholesterol efflux through LPL-mediated atherogenesis (S2 Fig and Fig 2B).

Compactin is used to inhibit 3-hydroxy-3-methylglutaryl coenzyme A reductase (HMG-CoA reductase), thus blocking cholesterol synthesis and making cells dependent on external cholesterol [27]. *L*. *acidophilus* K301-mediated *ABCA1* and *ABCG1* mRNA expression was significantly decreased by compactin in a dose-dependent manner (Fig 2C). In contrast, TO901317 did not decrease the mRNA levels of *ABCA1* and *ABCG1* in the compactin-treated cells (Fig 2D). These results suggest that *L*. *acidophilus* K301, but not TO901317, increases ABCA1-mediated cholesterol efflux through the HMG-CoA reductase pathway.

The production of mevalonate from HMG-CoA is catalyzed by HMG-CoA reductase and is involved in cholesterol synthesis (S3 Fig). To determine whether compactin-mediated inhibition of *ABCA1* and *ABCG1* gene expression in macrophages is specific to the inhibitory effect on HMG-CoA reductase, the restorative effect of mevalonate on the suppression of *ABCA1* and *ABCG1* gene expression by compactin was examined. As shown in Fig 2E, mevalonate treatment rescued the compactin-mediated reduction in *ABCA1* and *ABCG1* expression. Expression of these genes was not induced by mevalonate in the absence of *L*. *acidophilus* K301 and the addition of mevalonate alone had no significant effect on *ABCA1* and *ABCG1* mRNA expression level. The protein expression of ABCA1 and ABCG1 was also increased by co-treatment with mevalonate and *L*. *acidophilus* K301 (Fig 2F). *ABCA1* and *ABCG1* gene expression in macrophages treated with compactin and mevalonate increased in a *L*. *acidophilus* K301 dose-dependent manner (Fig 2G). These data suggest that *L*. *acidophilus* K301-mediated ABCA1 and ABCG1 expression is associated with intracellular cholesterol synthesis mediated by the mevalonate pathway, including the HMG-CoA reductase pathway. Thus, the *L*. *acidophilus* K301-mediated pathway is different from the TO901317-mediated cholesterol synthesis pathway.

### *L*. *acidophilus* K301 increased apoA-I mediated cholesterol efflux in macrophages

**Fig 3:**
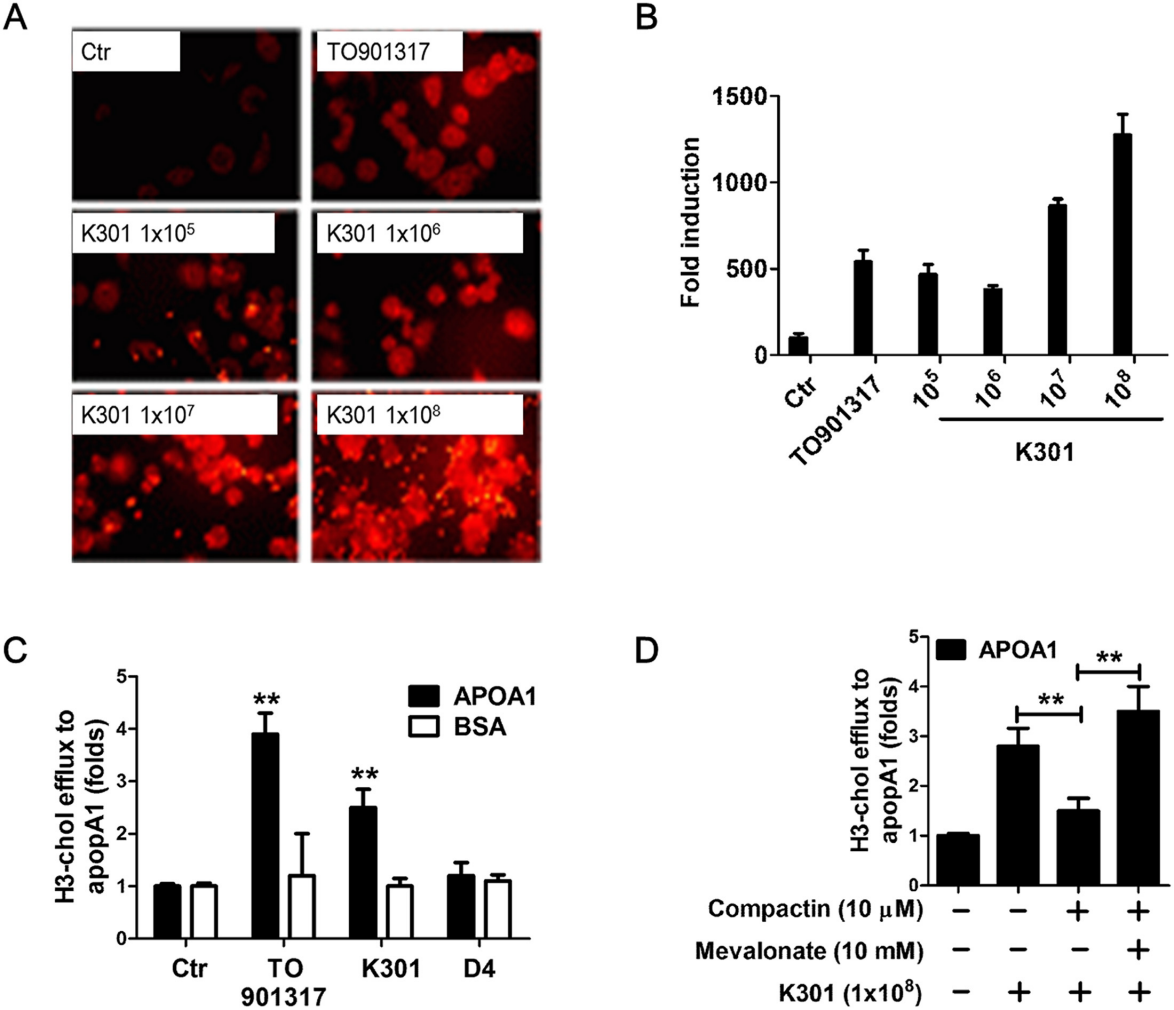
*L*. *acidophilus* K301 increased apoA-I mediated cholesterol efflux in macrophages. Cholesterol efflux from [H^3^]-cholesterol (1 μCi/ml)-loaded macrophages to apoA-I (10 μg/ml) was measured after incubation with heat-killed *L*. *acidophilus* K301 or TO901317. (A) The binding of Alexa-labeled apoA-I onto the surface of heat-killed *L*. *acidophilus* K301 treated-macrophages is shown. (B) Densitometry analysis was performed by ImageJ software after repeated experiments. The data shown is representative of two independent experiments. (C) Cholesterol efflux is expressed as the percentage of [H^3^]-cholesterol in the media relative to total [H^3^]-cholesterol (media and cells). An unpaired t-test was used to determine the significance of differences between treatment groups and control. (D) Cholesterol efflux was examined after stimulation with heat-killed *L*. *acidophilus* K301 in the presence or absence of compactin and mevalonate. Results are the means of three independent experiments (mean±S.D). **P < 0.01 vs. untreated cells. The error bars in individual samples are shown as variations from triplicate assays.

ABCA1 and ABCG1 directly interact with apoA-I and transfer cholesterol onto apoA-I in the cholesterol efflux mechanism [11]. The binding ability of apoA-I to *L*. *acidophilus* K301 treated-macrophage cells was examined. The binding of Alexa labeled apoA-I onto the surface of *L*. *acidophilus* K301 treated-macrophages increased in a dose-dependent manner (Fig 3A). Densitometric scanning of the immunofluorescence result displayed relative expression of apoA-I by *L*. *acidophilus* K301 treatment (Fig 3B). *L*. *acidophilus* K301 also increased the efflux of cholesterol up to 2.3-fold in apoA-I treated conditions, whereas no effect was observed in the absence of apoA-I (Fig 3C). TO901317 increased the cholesterol efflux 3.7-fold (p<0.01) in apoA-I treated conditions. In contrast, *L*. *rhamnosus* D4, which had no ability to induce ABCA1 and ABCG1 (Fig 1A), did not affect cholesterol efflux. When cells were treated with compactin, *L*. *acidophilus* K301-mediated cholesterol efflux was decreased and this effect was rescued by mevalonate treatment (Fig 3D). These data suggest that *L*. *acidophilus* K301 increases cholesterol efflux through the interaction of apoA-I and ABCA1 or ABCG1, which was increased by *L*. *acidophilus* K301.

### *L*. *acidophilus* K301 modulated gene expression related to reverse cholesterol transport by induction of squalene epoxidase and oxidosqualene cyclase

**Fig 4:**
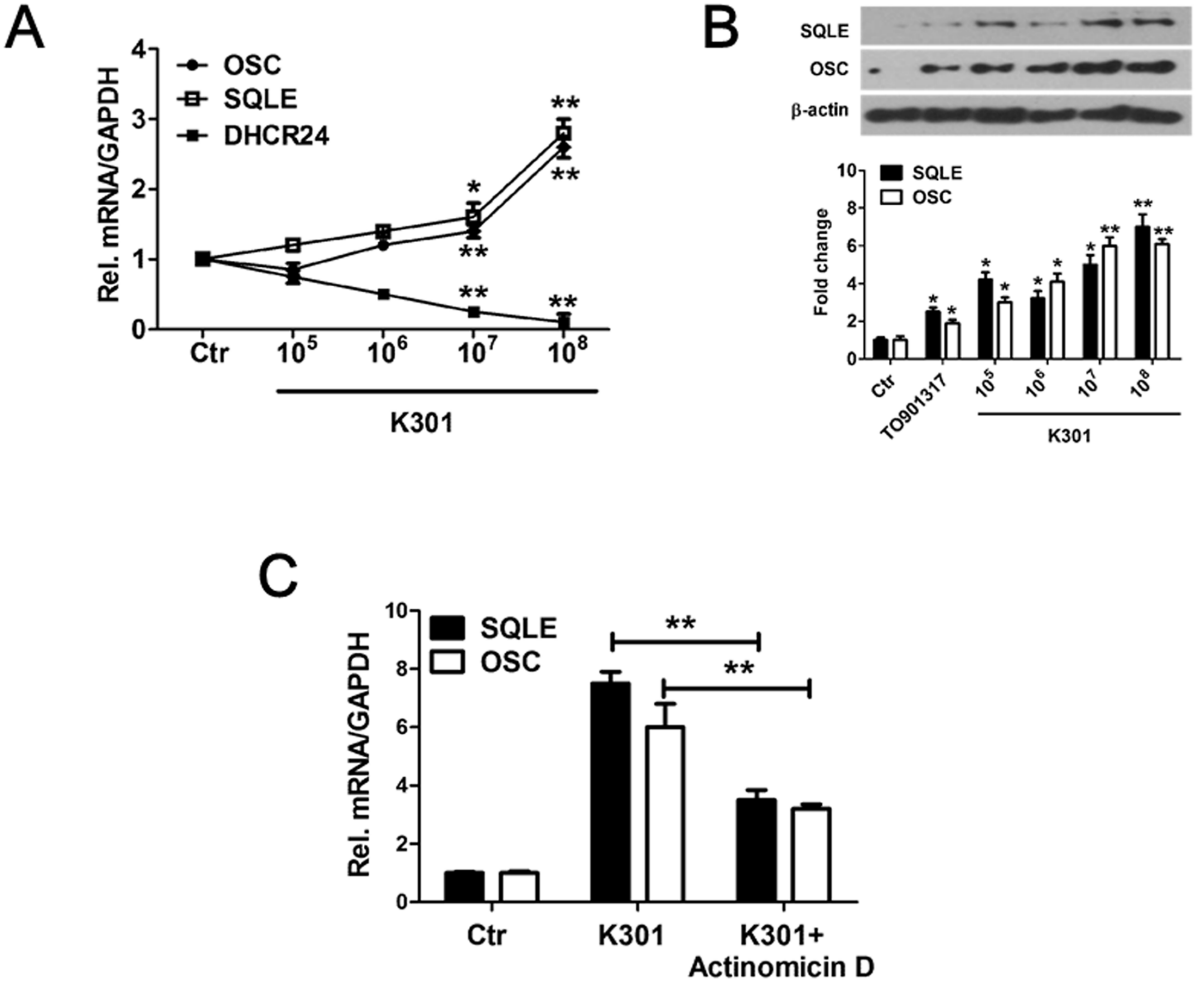
*L*. *acidophilus* K301 modulated reverse cholesterol transport-related gene expression by induction of squalene epoxidase and oxidosqualene cyclase. PMA-differentiated macrophages were stimulated with various concentrations of heat-killed *L*. *acidophilus* K301 for 12 h. The expression of SQLE and OSC was examined by real-time PCR (A) and western blotting using specific anti-SQLE and anti-OSC antibodies (B). The amount of protein expression was normalized to beta-actin and the fold change is shown (lower panel of B). (C) The mRNA expression of *AQLE* and *OSC* was examined in cells stimulated with heat-killed *L*. *acidophilus* K301 in the presence of Actinomycin D. mRNA level was normalized to *GAPDH*. Data are given as mean±SD. *P < 0.05; **P < 0.01 vs. control (Ctr). The error bars in individual samples are shown as variations from triplicate assays.

Squalene epoxidase (SQLE) and 2,3-oxidosqualene:lanosterol cyclase (OSC) are two key cholesterol biosynthesis enzymes. After treatment with *L*. *acidophilus* K301, levels of *SQLE* and *OSC* gene expression in THP-1 macrophages increased in a dose-dependent manner (Fig 4A). In contrast, expression of 24-dehydrocholesterol reductase (DHCR24), an enzyme that functions at the end of cholesterol biosynthesis, was decreased in *L*. *acidophilus* K301-treated cells. Protein expression of SQLE and OSC was consistent with the mRNA expression, indicating that *L*. *acidophilus* K301 enhances the synthesis of the oxysterol 24(S), 25-epoxychelesterol (24, 25-EC) in THP-1-differentiated macrophages (Fig 4B). When cells were treated with Actinomycin D plus *L*. *acidophilus* K301, which inhibits *ABCA1* and *ABCG1* mRNA expression, SQLE and OSC expression was inhibited compared to *L*. *acidophilus* K301 treatment only (Fig 4C). These findings suggest that *L*. *acidophilus* K301-induced ABCA1 and ABCG1 expression is associated with endogenous LXR ligands, such as 24, 25-EC, and the effect of *L*. *acidophilus* K301 on ABCA1 and ABCG1 may be partially dependent on LXR.

### *L*. *acidophilus* K301 induced ABCA1 and ABCG1 production through the endogenous LXR agonist, 24(*S*), 25-epoxcycholesterol

**Fig 5:**
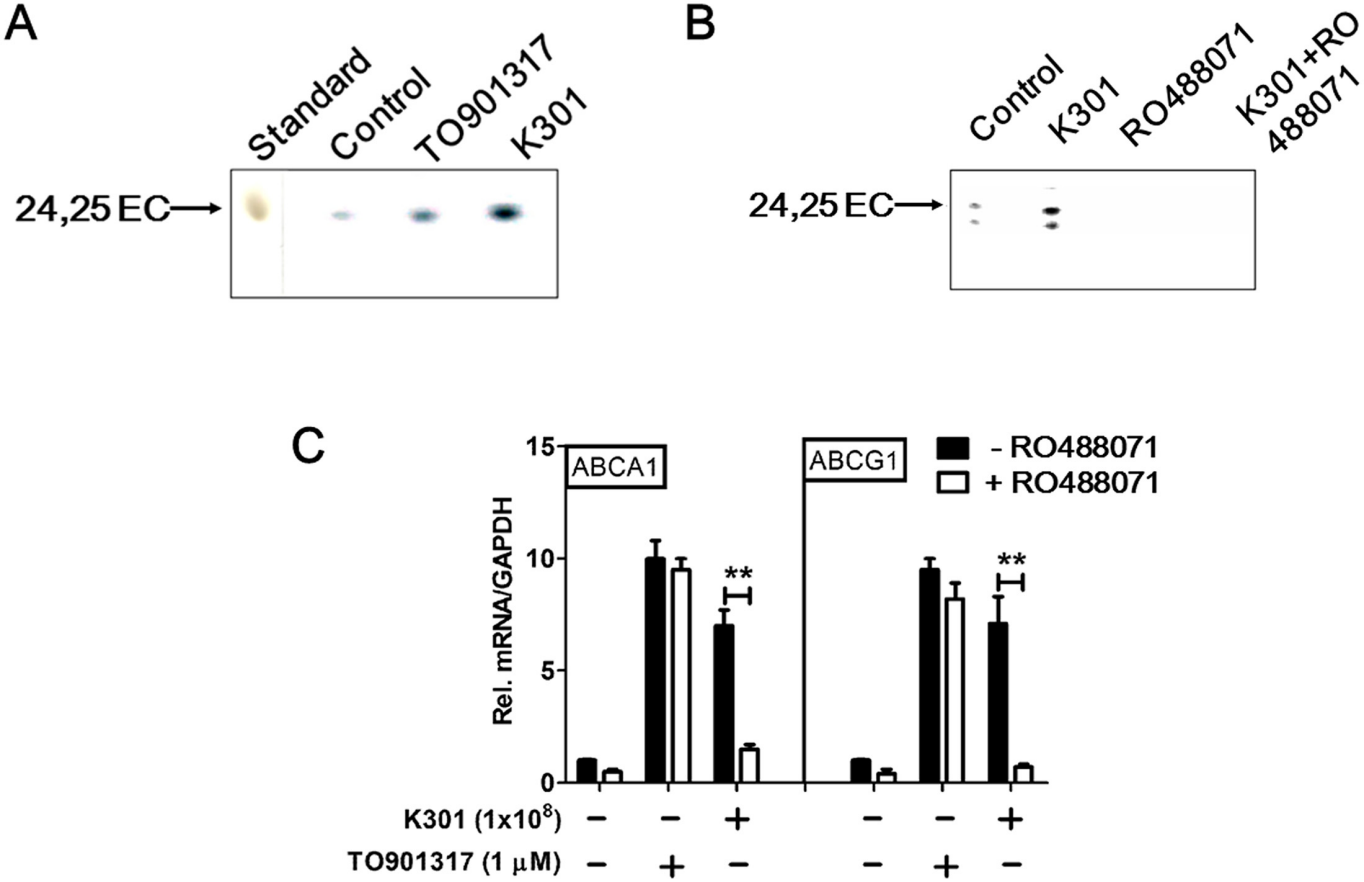
*L*. *acidophilus* K301 induced ABCA1 and ABCG1 production through the endogenous LXR agonist, 24(*S*), 25-epoxcycholesterol. (A) PMA-differentiated macrophages were incubated with TO901317 and heat-killed *L*. *acidophilus* K301 in medium containing [^14^C]-acetate for 16 h. Neutral lipid extracts were separated by TLC and visualized by exposure to X-ray film. (B) Heat-killed *L*. *acidophilus* K301-stimulated THP-1 macrophages were treated with a specific inhibitor of endogenous ligand synthesis, RO 488071, and neutral lipid extracts were separated using TLC. (C) Heat-killed *L*. *acidophilus* K301-stimulated THP-1 macrophages were treated with RO 488071 and mRNA levels of *ABCA1* and *ABCG1* were determined using real-time PCR. mRNA levels were normalized to *GAPDH*. Data are given as means ±SD. **P < 0.01 vs. untreated cells. The error bars in individual samples are shown as variations from triplicate assays.

The endogenous regulator 24(S), 25-epoxycholesterol (24, 25-EC) decreases cholesterol synthesis by interfering with DHCR24, resulting in rapid accumulation of the substrate desmosterol [28]. 24, 25-EC is produced in a shunt of the cholesterol synthetic pathway and works at multiple points to maintain cellular cholesterol homeostasis. 24, 25-EC reduces cell cholesterol levels through inhibition of cholesterol synthesis by stimulating HMGR degradation, increased uptake by suppressing SREBP activation, or the acceleration of export by serving as a potent ligand for LXR [29]. It is also known that endogenous synthesis of 24, 25-EC increases *ABCA1* and *ABCG1* gene expression [13]. In this study, we examined whether *L*. *acidophilus* K301 activates the shunt pathway for cholesterol efflux. Analysis by thin-layer chromatography (TLC) showed the induction of 24, 25-EC production in *L*. *acidophilus* K301-treated (1×10^8^ cells/ml) macrophages (Fig 5A), which was suppressed by treatment with RO 488071 (an inhibitor of oxidosqualene cyclase [OSC]) (Fig 5B). Treatment with RO 488071 also significantly reduced the *L*. *acidophilus* K301-mediated induction of ABCA1 and ABCG1, indicating that OSC is involved in the *L*. *acidophilus* K301-mediated induction of ABCA1 and ABCG1 (Fig 5C). Together, these data suggest that increased cholesterol efflux is mediated by induction of 24, 25-EC, an endogenous LXR agonist, in the shunt pathway.

### *L*. *acidophilus* K301 increased the gene expression of *ABCA1*, *ABCG1*, and *OSC* in mouse primary macrophages

**Fig 6:**
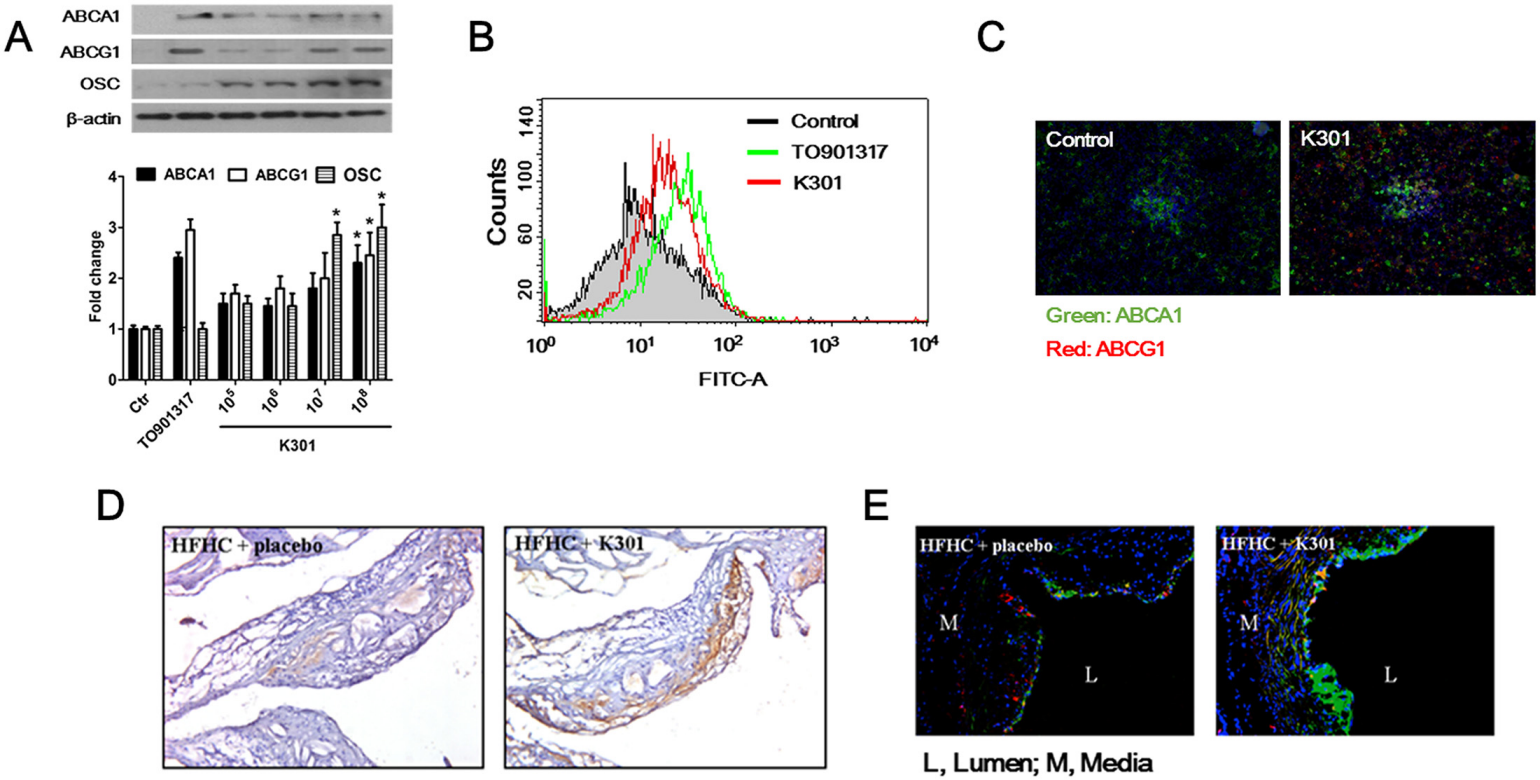
*L*. *acidophilus* K301 increased gene expression of *ABCA1*, *ABCG1*, and *OSC* in mouse primary macrophages. (A) Mouse peritoneal macrophages isolated from *ApoE*^*-/-*^ mice were incubated with heat-killed *L*. *acidophilus* K301 for 24 h and the expression of ABCA1, ABCG1, and OSC was examined by western blotting using specific antibodies. The amount of protein expression was normalized to that of β-actin. Densitometry analysis was performed by ImageJ software after repeated experiments (lower panel). The error bars in individual samples are shown as variations from triplicate assays. (B) *ApoE*^*-/-*^ mice were intraperitoneally injected with 1×10^11^/kg heat-killed *L*. *acidophilus* K301 or TO901317. At 48 h after injection, macrophages were isolated and the expression of ABCA1 was examined by flow cytometric analysis. (C) The expression of ABCA1 and ABCG1 in macrophages isolated from heat-killed *L*. *acidophilus* K301-injected *ApoE*^*-/-*^ mice was visualized by immunofluorescence staining. (D) Representative immunostaining images showing the change in expression of ABCA1 in *ApoE*^*-/-*^ mice fed a high-fat high-cholesterol (HFHC) diet or treated with heat-killed *L*. *acidophilus* K301. (E) Immunostaining images of SQLE and OSC in HFHC-fed *ApoE*^*-/-*^ mice and *L*. *acidophilus* K301-infused *ApoE*^*-/-*^ mice; typical images from the area inside the plaques in each group are displayed. In all color images, DAPI (DNA) is shown as blue, whereas target proteins are shown as green (OSC) or red (SQLE). Data shown is representative of three independent experiments.

To examine the effect of *L*. *acidophilus* K301 in mouse macrophages, mouse peritoneal macrophages were treated with *L*. *acidophilus* K301. Similar to the results in human macrophages, gene expression of *ABCA1*, *ABCG1*, and *OSC* was induced by *L*. *acidophilus* K301 treatment in mouse macrophages in a dose-dependent manner (Fig 6A). In an *ex-vivo* study, *ApoE*^*-/-*^ mice were intraperitoneally injected with *L*. *acidophilus* K301 and the expression of ABCA1 and ABCG1 was examined by flow cytometry (Fig 6B) and immunofluorescence (Fig 6C) analysis of macrophages isolated 48 h after injection with *L*. *acidophilus* K301 (1×10^11^/kg). In both experiments, ABCA1 and ABCG1 expression was increased by *L*. *acidophilus* K301 compared to the control.

Next, the expression of ABCA1, SQLE, and OSC in the aortic sinus was examined. Mice treated with *L*. *acidophilus* K301 after high-fat high-cholesterol (HFHC) treatment showed increased expression of ABCA1 compared to mice with HFHC treatment only (Fig 6D). Induction of SQLE and OSC was also observed in the aortic sinus, indicating that synthesis of 24, 25-EC in the artery was induced by *L*. *acidophilus* K301 infusion (Fig 6E).

### Administration of *L*. *acidophilus* K301 prevented atherosclerotic lesion development

**Fig 7:**
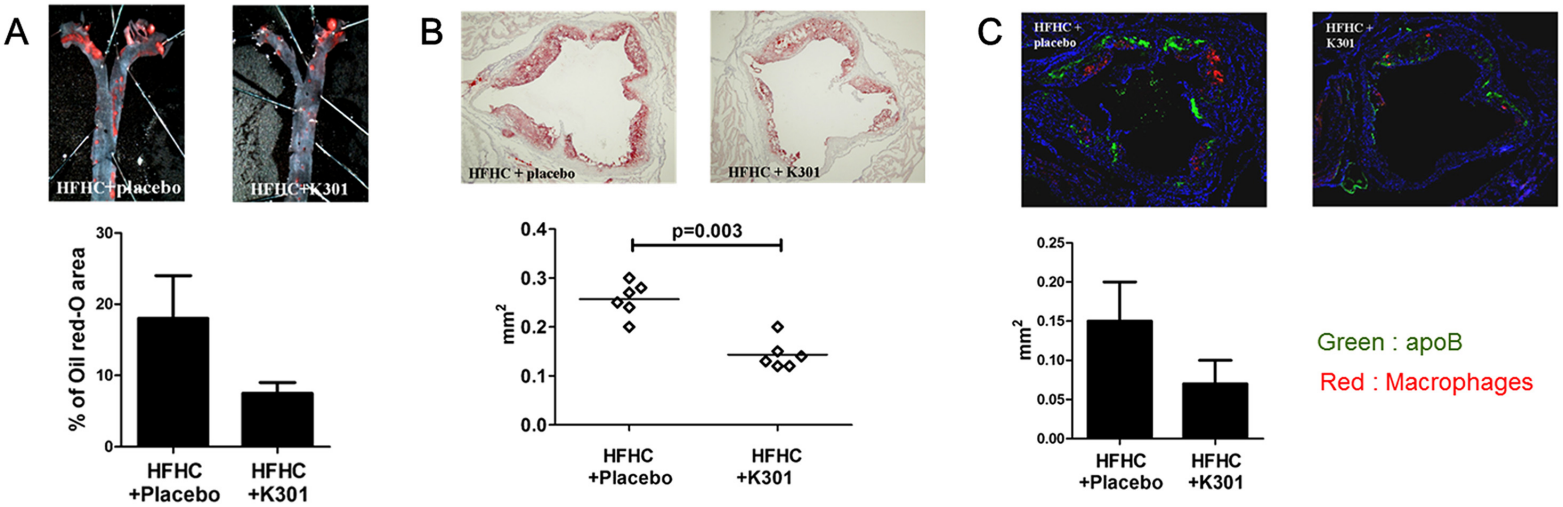
Administration of *L*. *acidophilus* K301 prevented atherosclerotic lesion development. *ApoE*^*-/-*^ mice were treated with heat-killed *L*. *acidophilus* K301 and/or a high-fat high-cholesterol (HFHC) diet for 8 weeks. (A) Representative en face Oil Red-O stained aortas from *ApoE*^*-/-*^ mice fed an HFHC diet and co-treated daily with 1×10^13^/kg heat-killed *L*. *acidophilus* K301 for 8 weeks is shown, and the percentage of Oil Red-O stained area was calculated (lower panel). (B) Oil Red-O stained areas of aortic sinus are shown with quantitation by computer-assisted morphometery (lower panel). All results are shown as mean ± S.D. (C) Accumulation of ApoB-100 and macrophages in HFHC-fed *ApoE*^*-/-*^ mice and heat-killed *L*. *acidophilus* K301-infused *ApoE*^*-/-*^ mice was examined by immunofluorescence analysis. ApoB and macrophages from the area inside the plaques in each group are displayed. In all color images, DAPI is shown in blue, and target proteins in green (ApoB-100) or red (macrophages). Data shown is representative of three independent experiments.

Serum profiles were measured in the 16th week (after 8 weeks on HFHC diet and 8 weeks on a normal chow diet). Table 1 shows the effects of dietary *L*. *acidophilus* K301 on serum cholesterol levels in *ApoE*^*-/-*^ mice. HDL concentrations in the *L*. *acidophilus* K301-treated group were significantly increased compared with the HFHC diet group. Glutamic oxaloacetic transaminase (GOT) and gamma-glutamic pyruvic transaminase (GTP) concentrations did not show a significant difference between the groups. These results suggest that *L*. *acidophilus* K301 increased serum HDL levels, but decreased total cholesterol levels, in high-fat high-cholesterol fed *ApoE*^*-/-*^ mice. Oil Red O staining was performed to evaluate cholesterol uptake. The Oil Red O stained area of the en face view of aorta specimen was 18.1 ± 5.6% in the reference group treated with HFHC only and was decreased by 42.3% in the K301-treated group (*P* < 0.05) (Fig 7A). The Oil Red O stained area in the aortic sinus of *ApoE*^*-/-*^ mice was decreased by 60.2% in the *L*. *acidophilus* K301-treated group compared with the reference group at 16 weeks (Fig 7B), indicating that administration of *L*. *acidophilus* K301 prevented atherosclerotic lesion development by inhibiting lipid accumulation in the aorta of HFHC-fed *ApoE*^*-/-*^ mice. Staining of MOMA-2, a specific marker of mouse macrophages, revealed that the area of macrophage infiltration was 147.3 ± 38.4 μm^2^ in the HFHC diet only group. The MOMA-2 stained area decreased by 49.1% (72.3 ± 39.5 μm^2^) in the *L*. *acidophilus* K301-treated group compared with the HFHC-only group at 16 weeks (*P* < 0.05) (Fig 7C). These data suggest that *L*. *acidophilus* K301 inhibited macrophage infiltration in atherosclerotic lesions of HFHC-fed *ApoE*^*-/-*^ mice.

**Table 1:** 

Group	n	TC (mg/dL)	TG (mg/dL)	HDL(mg/dL)	GOT(U/L)	GPT (U/L)
HFHC	4	865±121.66	58.33±10.41	23.33±2.89	105±27.83	43.33±2.89
K301	5	530±55.79*	75.00±12.24	48.75±16.00	100±9.35	47.00±5.70

Data were expressed as mean ± S.D. GOT, glutamic oxaloacetic transaminase; GPT, gamma-glutamic pyruvic transaminase; HDL, high-density lipoprotein; TC, total cholesterol; TG, triglyceride.

* *P* = 0.001544

¶ *P* = 0.045144

## Discussion

Administration of fermented dairy products containing LAB has shown the potential to reduce serum cholesterol levels. Several possible mechanisms for LAB-mediated cholesterol removal are absorption of cholesterol by growing cells, binding of cholesterol to the cellular surface, incorporation of cholesterol into the cellular membrane, deconjugation of bile by bile salt hydrolase, coprecipitation of cholesterol with deconjugated bile, binding of bile by fiber, and production of short-chain fatty acids by oligosaccharides [30]. In particular, it is known that *lactobacillus* species have the ability to reduce cholesterol content. For example, the probiotic bacteria *L*. *paracasei* possess cholesterol lowering activity, and LPS-mediated IL-1β protein expression in alveolar macrophages (AMs) from pigs fed a high-fat diet with probiotic bacteria (HFPB diet) was significantly lower than that in AMs from pigs fed the HF diet only [31]. Huang and colleagues demonstrated that *L*. *acidophilus* ATCC 4356 prevents atherosclerosis by inhibition of intestinal cholesterol absorption in *ApoE*^*-/-*^ mice [32]. The probiotics *L*. *rhamnosus* BFE5264 and *L*. *plantarum* NR74 also promote cholesterol efflux and inhibit expression of inflammatory cytokines such as IL-1β and TNF-α, which are increased by LXR activation [33]. In particular, these two strains activated LXR and induced cholesterol efflux by promoting the expression of ABCA1 and ABCG1. Kaplan and colleagues have reported that bacterial endotoxin increases the expression of ABCA1, but not ABCG1, through a LXR-independent pathway [34]. However, there are no clues to how LAB modulates the ABCA1- and ABCG1-mediated induction of cholesterol efflux.

This study demonstrated the effects of different LAB strains on the induction of genes that are regulated by LXR in human and mouse macrophages. Some LAB species seem to increase *ABCA1* and *ABCG1* gene expression, which is necessary to induce intracellular cholesterol synthesis. Pretreatment with compactin, a HMG-CoA reductase inhibitor, prior to *L*. *acidophilus* K301 stimulation inhibited the expression of ABCA1 and ABCG1 in the macrophages. This result indicates that *L*. *acidophilus* K301 induces ABCA1 and ABCG1 production through an endogenous LXR agonist, such as 24(*S*), 25-epoxcycholesterol and/or 22(*R*)-hydroxycholesterol, in intracellular cholesterol synthetic pathways, suggesting that *L*. *acidophilus* K301 induces the expression of ABCA1 and ABCG1 via a LXR-dependent pathway. Furthermore, incubation with mevalonate rescued the compactin-mediated reduction of ABCA1 and ABCG1 expression in *L*. *acidophilus* K301-stimulated macrophages. The induction of ABCA1 and ABCG1 by *L*. *acidophilus* K301 was established by an increase in the level of endogenous LXR agonist, 24, 25-EC, which resulted from the induction of SQLE and OSC expression. Treatment of macrophages with RO 488071, an OSC inhibitor, inhibited the expression of ABCA1 and ABCG1. These results indicate that *L*. *acidophilus* K301 induced production of both ABCA1 and ABCG1 through 24, 25-EC synthesized by the activation of SQLE and OSC.

These findings imply that heat-killed LAB, especially *L*. *acidophilus*, might modulate the cholesterol level in macrophages through RCT. Furthermore, this transport increases the level of HDL in blood and reduces foam cell formation in cholesterol-loaded macrophages, which is known to have a beneficial effect in atherosclerosis. Thus, *L*. *acidophilus* K301 is a potential probiotic strain for application in the prevention of atherosclerosis via cholesterol reverse transport. Any concerns regarding the number of live microorganisms can be allayed by the fact that heat-killed LAB maintained the ability to induce RCT. It is possible that another unknown mechanism might also be involved, such as a critical ligand or transcription regulator related to LXR signaling or cholesterol synthesis mechanisms. These possibilities will be tested in the next phase of our experiment.

The physiological switch of macrophage may occur during atherosclerosis. Macrophages gradually accumulate in atherosclerotic lesions, which is enriched in LDL and extracellularmatrix proteoglycans, and adhere to the areas of tunica intima. As atherosclerotic lesions progress, macrophages produce pro-inflammatory cytokines and that is the reason atherosclerosis is considered to be an inflammatory disease [35]. The activation of M1 macrophages is induced by bacterial cell wall components including LPS and LTA, and they are characterized with pro-inflammatory cytokine secretion. On the other hand, IL-10 producing macrophages are considered as M2 activation [36]. In the current study, we examined the expression of TNF-α and IL-10 from PMA-differentiated THP-1 cells and peritoneal macrophages from mice using lipopolysaccharide (LPS) and *L*. *acidophilus* K301. When THP-1 and peritoneal cells were treated with LPS, TNF-α was significantly increased in both cells, while pretreatment of *L*. *acidophilus* K301 followed by LPS retreatment led to decrease TNF-α expression (S4 Fig). These results suggest that probiotics including *L*. *acidophilus* K301 have an anti-inflammatory effect, which may alleviate atherosclerotic plaque formation. On the other hand, IL-10 production was not significantly altered in both cells (data not shown). Macrophages seemed to be switched into M1 macrophages by LPS stimulation, and they lost M1 activation state by *L*. *acidophilus* K301 pretreatment. More studies are needed to prove the activation state of macrophages by bacterial cell wall components.

In conclusion, elevated serum cholesterol causes atherosclerosis and coronary heart disease. LAB are known to remove cholesterol through precipitation with bile, incorporation into the LAB membrane, or absorption by cells. The current study identified that *L*. *acidophilus* K301 can regulate the expression of genes related to cholesterol reverse transport via the induction of endogenous LXR agonist. *L*. *acidophilus* K301 increased 24, 25 EC production by induction of SQLE and OSC, which activate LXR and the LXR-related genes *ABCA1* and *ABCG1*. ABCA1 and ABCG1 increase cholesterol efflux through interaction with apoA-I. In addition, *L*. *acidophilus* K301 seems to inhibit cholesterol biosynthesis by degrading HMGR. Thus, our data suggest that *L*. *acidophilus* K301 has therapeutic potential as an anti-atherosclerotic agent.

## Supporting Information

S1 Fig mRNA expression profiles of three monocyte-macrophage maturation markers. (A) THP-1 cells were differentiated for 0–48 h using 100 mM PMA. mRNA level of CD36 (solid), CCR2 (open square), and CD11b (open triangle) are presented. (B) PMA differentiated THP-1 cells were incubated with 50 μg/ml AcLDL. Lipid accumulation was visualized by Oil Red-O staining and microscopy. Data show means ± SD (N = 3).
(TIF)

S2 Fig LXR-related gene expression. PMA-differentiated macrophages were incubated with 1 μM TO9013 or 1×10^7^ heat-killed *L*. *acidophilus* K301 for 16 h and then total RNA was isolated. Expression of LXR-related genes was analyzed using an Affymetrix GeneChip (Affymetrix, Santa Clara, CA).
(TIF)

S3 Fig Schematic overview of *L*. *acidophilus* K301-mediated cholesterol efflux. *L*. *acidophilus* K301 induces *SQLE* and *OSC* gene expression, which results in the induction of 24, 25-EC in the shunt pathway. 24, 25-EC activates LXR and LXR-related genes *ABCA1* and *ABCG1*, while decreasing expression of *LPS*. *L*. *acidophilus* K301 inhibits cholesterol biosynthesis by degrading HMGR.
(TIF)

S4 Fig LPS-mediated TNF-α production was inhibited by *L*. *acidophilus* K301. PMA-differentiated THP-1 cells and peritoneal macrophages from mouse were pre-treated with or without *L*. *acidophilus* K301 for 18 h, and then 0.1 μg/ml LPS was re-treated for 4 h. TNF-α expression was examined by sandwich ELISA method (R&D Systems, MN, USA) using culture supernatants. The error bars in individual samples are shown as variations from triplicate assays.
(TIF)

S1 Table Real-time PCR primers used in this study. (DOCX)

S2 Table Abbreviations of bacterial strains used in this study. (DOCX)

## References

[1] Kannel WB, Doyle JT, Ostfeld AM, Jenkins CD, Kuller L, Podell RN, et al (1984) Optimal resources for primary prevention of artherosclerotic diseases. Atherosclerosis Study Group. Circulation 70:155A–205A. 6398769

[2] Ross R (1993) The pathogenesis of atherosclerosis: A perspective for 1990s. Nature 362: 801–809. 8479518 10.1038/362801a0

[3] Law MR, Wald NJ, Wu T, Hackshaw Q, Baily A (1994) Systematic underestimation of association between serum cholesterol concentration and ischemic heart disease in observation studies: Data from BUPA study. Br Med J 308: 363–366. 8124143 10.1136/bmj.308.6925.363 PMC2539480

[4] Slalter J, Chaplin M, Dicjerson J, Davis F (1996) Bile acid and health: Is fiber the answer? Nur Food Sci 96: 29–33.

[5] Suckling KE, Benson GM, Bond B, Gee A, Glen A, Haynes C, et al (1991) Cholesterol lowering and bile acid excretion in the hamster with cholestyramine treatment. Atherosclerosis 89: 183–190. 1793446 10.1016/0021-9150(91)90059-c

[6] Erkelens DW, Baggen MG, Van Doormaal JJ, Kettner M, Koningsberger JC, Mol MJ (1998) Clinical experience with simvastatin compared with cholestyramine. Drugs 36: 87–90. 10.2165/00003495-198800363-00018 3254824

[7] Cuchel M, Rader DJ (2006) Macrophage reverse cholesterol transport: key to the regression of atherosclerosis? Circulation 113: 2548–2555. 16735689 10.1161/CIRCULATIONAHA.104.475715

[8] Goldstein JL, DeBose-Boyd R, Brown MS (2006) Protein sensors for membrane sterols. Cell 124: 35–46. 16413480 10.1016/j.cell.2005.12.022

[9] Gill S, Chow R, Brown AJ (2008) Sterol regulators of cholesterol homeostasis and beyond: the oxysterol hypothesis revisited and revised. Prog Lipid Res 47: 391–404. 10.1016/j.plipres.2008.04.002 18502209

[10] Terasaka N, Hiroshima A, Koieyama T, Ubukata N, Morikawa Y, Nakai D, et al (2003) TO-901317, a synthetic liver X receptor ligand, inhibits development of atherosclerosis in LDL receptor-deficient mice. FEBS Lett 536: 6–11. 12586329 10.1016/s0014-5793(02)03578-0

[11] Tontonoz P, Mangelsdorf DJ (2003) Liver X Receptor Signaling Pathways in Cardiovascular Disease. Mol Endocrinol 17: 985–993. 12690094 10.1210/me.2003-0061

[12] Lehrke M, Lebherz C, Millington SC, Guan HP, Millar J, Rader DJ, et al (2005) Diet-dependent cardiovascular lipid metabolism controlled by hepatic LXR alpha. Cell Metab 1: 297–308. 16054077 10.1016/j.cmet.2005.04.005

[13] Beyea MM, Heslop CL, Sawyez CG, Edwards JY, Markle JG, Hegele RA, et al (2007) Selective up-regulation of LXR-regulated genes ABCA1, ABCG1, and APOE in macrophages through increased endogenous synthesis of 24(S),25-epoxycholesterol. J Biol Chem 282: 5207–5216. 17186944 10.1074/jbc.M611063200

[14] Kim JY, Kim H, Jung BJ, Kim NR, Park JE, Chung DK (2013) Lipoteichoic acid isolated from *Lactobacillus plantarum* suppresses LPS-mediated atherosclerotic plaque inflammation. Mol Cells 35: 115–124. 10.1007/s10059-013-2190-3 23456333 PMC3887899

[15] Kim H, Jung BJ, Jeong J, Chun H, Chung DK (2014) Lipoteichoic acid from *Lactobacillus plantarum* inhibits the expression of platelet-activating factor receptor induced by *Staphylococcus aureus* lipoteichoic acid or Escherichia coli lipopolysaccharide in human monocyte-like cells. J Microbiol Biotechnol 24: 1051–1058. 24786530 10.4014/jmb.1403.03012

[16] Jeong JH, Jang S, Jung BJ, Jang KS, Kim BG, Chung DK, et al (2015) Differential immune-stimulatory effects of LTAs from different lactic acid bacteria via MAPK signaling pathway in RAW 264.7 cells. Immunobiology 220: 460–466. 10.1016/j.imbio.2014.11.002 25433634

[17] De Smet I, Van Hoorde L, De Sayer N, Vande Woestyne M, Verstraete W (1994) In vitro study of bile salt hydrolase (BSH) activity of BSH isogenic *Lactobacillus plantarum* 80 strain and estimation of cholesterol lowering through enhanced BSH activity. Microb Ecol Health Dis 7: 315–329.

[18] Noh DO, Kim SH, Gilliland SE (1997) Incorporation of cholesterol into the cellular membrane of *Lactobacillus acidophilus* ATCC 4321. J Dairy Sci 80: 3107–3113. 9436091 10.3168/jds.S0022-0302(97)76281-7

[19] Usman HA (2001) Hypocholesterolemic effect of *Lactobacillus gasseri* SBTO270 in rats fed cholesterol enriched diet. J Dairy Res 68: 617–624. 11928958 10.1017/s0022029901005179

[20] Gilliland SE, Nelson CR, Maxwell CV (1985) Assimilation of cholesterol by *Lactobacillus acidophilus*. Appl Environ Microbiol 49: 377–381. 3920964 10.1128/aem.49.2.377-381.1985 PMC238411

[21] Gilliland SE, Speck ML (1997) Deconjugation of bile acids by intestinal *lactobacilli*. Appl Environ Microbiol 33: 15–18. 10.1128/aem.33.1.15-18.1977 PMC170567 13710

[22] Nakaya K, Ayaori M, Hisada T, Sawada S, Tanaka N, Iwamoto N, et al (2007) Telmisartan enhances cholesterol efflux from THP-1 macrophages by activating PPARgamma. J Atheroscler Thromb 14: 133–141. 17587765 10.5551/jat.14.133

[23] Wong J, Quinn CM, Brown AJ (2004) Statins inhibit synthesis of an oxysterol ligand for the liver x receptor in human macrophages with consequences for cholesterol flux. Arterioscler Thromb Vasc Biol 24: 2365–2371. 15514210 10.1161/01.ATV.0000148707.93054.7d

[24] Murthy S, Born E, Mathur SN, Field FJ (2002) LXR/RXR activation enhances basolateral efflux of cholesterol in CaCo-2 cells. J Lipid Res 43: 1054–1064. 12091489 10.1194/jlr.m100358-jlr200

[25] Kawashima RL, Medh JD (2014) Down-regulation of lipoprotein lipase increases ABCA1-mediated cholesterol efflux in THP-1 macrophages. Biochem Biophys Res Commun 450: 1416–1421. 10.1016/j.bbrc.2014.07.015 25017912 PMC4142826

[26] Schoonjans K, Gelman L, Haby C, Briggs M, Auwerx J (2000) Induction of LPL gene expression by sterols is mediated by a sterol regulatory element and is independent of the presence of multiple E boxes. J Mol Biol 304: 323–334. 11090277 10.1006/jmbi.2000.4218

[27] Goldstein JL, Helgeson JA, Brown MS (1979) Inhibition of cholesterol synthesis with compactin renders growth of cultured cells dependent on the low density lipoprotein receptor. J Biol Chem 254: 5403–5409. 221469

[28] Zerenturk EJ, Kristiana I, Gill S, Brown AJ (2012) The endogenous regulator 24(S),25-epoxycholesterol inhibits cholesterol synthesis at DHCR24 (Seladin-1). Biochim Biophys Acta 1821: 1269–1277. 10.1016/j.bbalip.2011.11.009 22178193

[29] Brown AJ (2009) 24(S),25-epoxycholesterol: a messenger for cholesterol homeostasis. Int J Biochem Cell Biol 41: 744–747. 10.1016/j.biocel.2008.05.029 18725318

[30] Kumar M, Nagpal R, Kumar R, Hemalatha R, Verma V, Kumar A, et al (2012) Cholesterol-lowering probiotics as potential biotherapeutics for metabolic diseases. Exp Diabetes Res 2012: 902917 10.1155/2012/902917 22611376 PMC3352670

[31] Trasino SE, Dawson HD, Urban JF Jr, Wang TT, Solano-Aguilar G (2013) Feeding probiotic *Lactobacillus paracasei* to Ossabaw pigs on a high-fat diet prevents cholesteryl-ester accumulation and LPS modulation of the Liver X receptor and inflammatory axis in alveolar macrophages. J Nutr Biochem 24: 1931–1939. 10.1016/j.jnutbio.2013.06.001 24060267

[32] Huang Y, Wang J, Quan G, Wang X, Yang L, Zhong L (2014) *Lactobacillus acidophilus* ATCC 4356 prevents atherosclerosis via inhibition of intestinal cholesterol absorption in apolipoprotein E-knockout mice. Appl Environ Microbiol 80: 7496–7504. 10.1128/AEM.02926-14 25261526 PMC4249224

[33] Yoon HS, Ju JH, Lee JE, Park HJ, Lee JM, Shin HK, et al (2013) The probiotic *Lactobacillus rhamnosus* BFE5264 and *Lactobacillus plantarum* NR74 promote cholesterol efflux and suppress inflammation in THP-1 cells. J Sci Food Agric 93: 781–787. 10.1002/jsfa.5797 22806829

[34] Kaplan R, Gan X, Menke JG, Wright SD, Cai TQ (2002) Bacterial lipopolysaccharide induces expression of ABCA1 but not ABCG1 via an LXR-independent pathway. J Lipid Res 43: 952–959. 12032171

[35] Mosser DM, Edwards JP (2008) Exploring the full spectrum of macrophage activation. Nat Rev Immunol. 8: 958–969. 10.1038/nri2448 19029990 PMC2724991

[36] Rőszer T (2015) Understanding the Mysterious M2 Macrophage through Activation Markers and Effector Mechanisms. Mediators Inflamm 2015:816460 10.1155/2015/816460 26089604 PMC4452191

