# Supplementary figures and images for: Lactobacillus acidophilus K301 Inhibits Atherogenesis via Induction of 24 (S), 25-Epoxycholesterol-Mediated ABCA1 and ABCG1 Production and Cholesterol Efflux in Macrophages
Source: PLoS One. 2016 Apr 27;11(4):e0154302. doi: 10.1371/journal.pone.0154302 (PMC4847857; doi:10.1371/journal.pone.0154302)

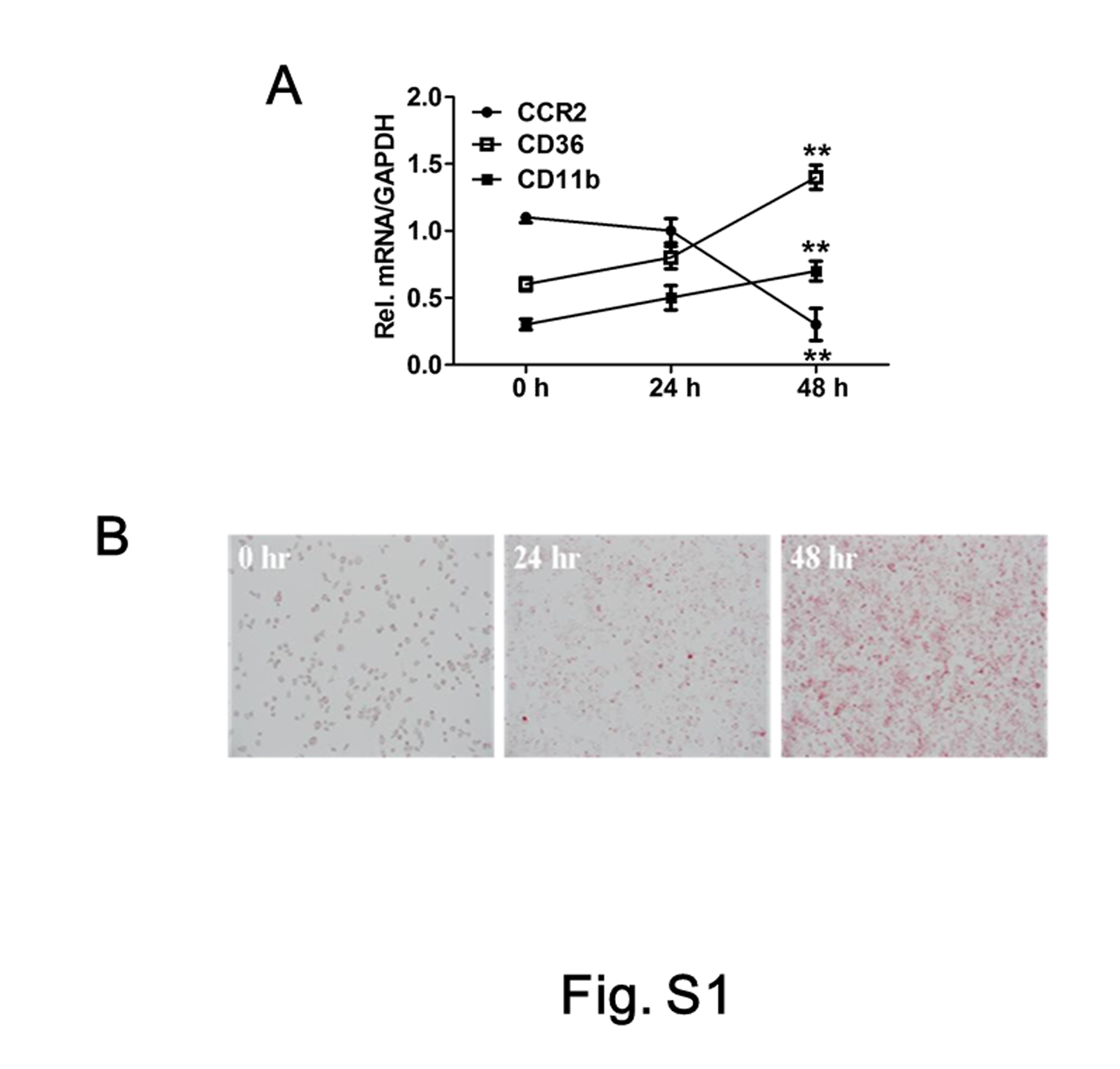

Supplement: S1 Fig — (A) THP-1 cells were differentiated for 0–48 h using 100 mM PMA. mRNA level of CD36 (solid), CCR2 (open square), and CD11b (open triangle) are presented. (B) PMA differentiated THP-1 cells were incubated with 50 μg/ml AcLDL. Lipid accumulation was visualized by Oil Red-O staining and microscopy. Data show means ± SD (N = 3). (TIF) [file pone.0154302.s001.TIF]

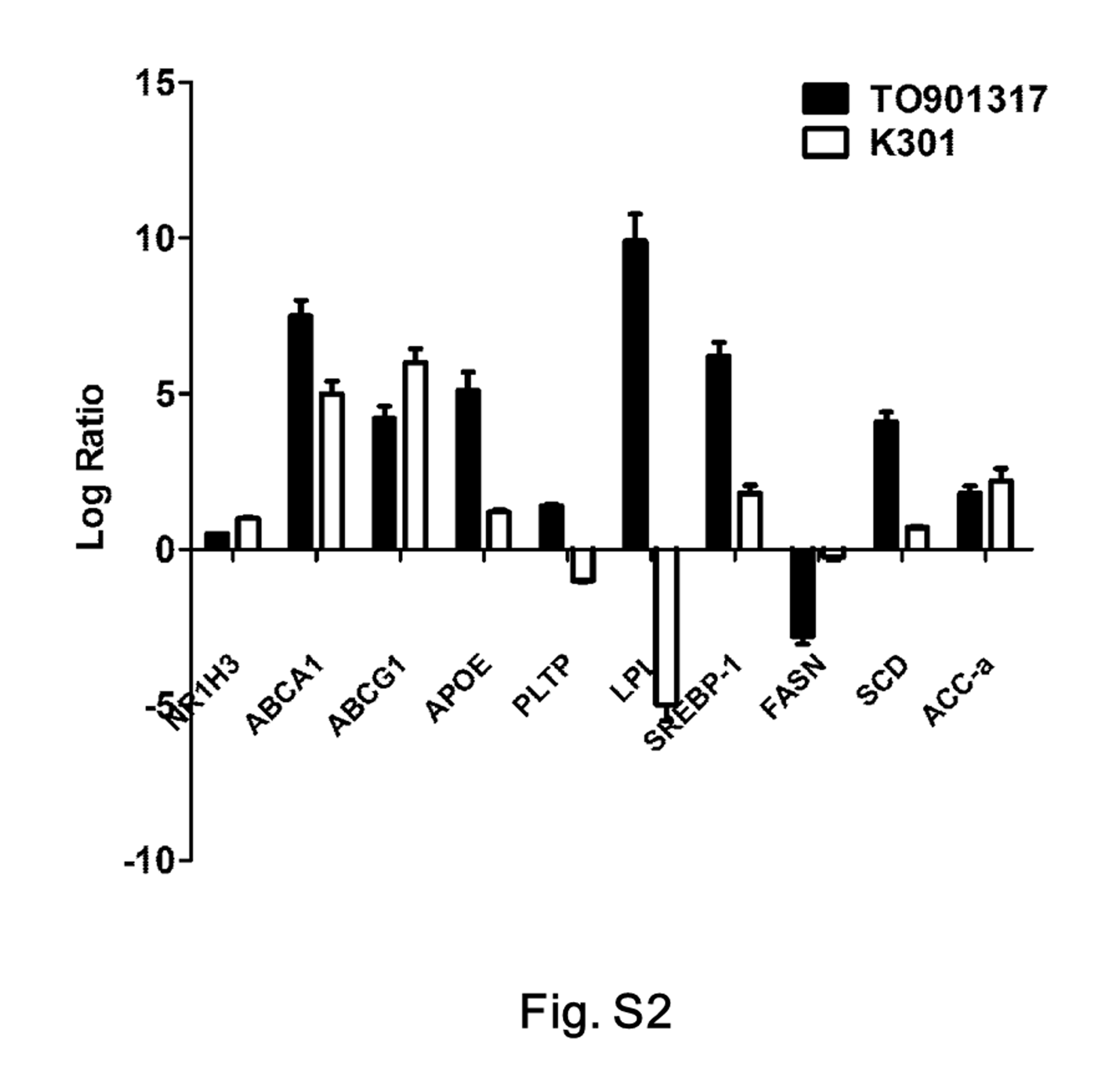

Supplement: S2 Fig — PMA-differentiated macrophages were incubated with 1 μM TO9013 or 1×10^7^ heat-killed L. acidophilus K301 for 16 h and then total RNA was isolated. Expression of LXR-related genes was analyzed using an Affymetrix GeneChip (Affymetrix, Santa Clara, CA). (TIF) [file pone.0154302.s002.TIF]

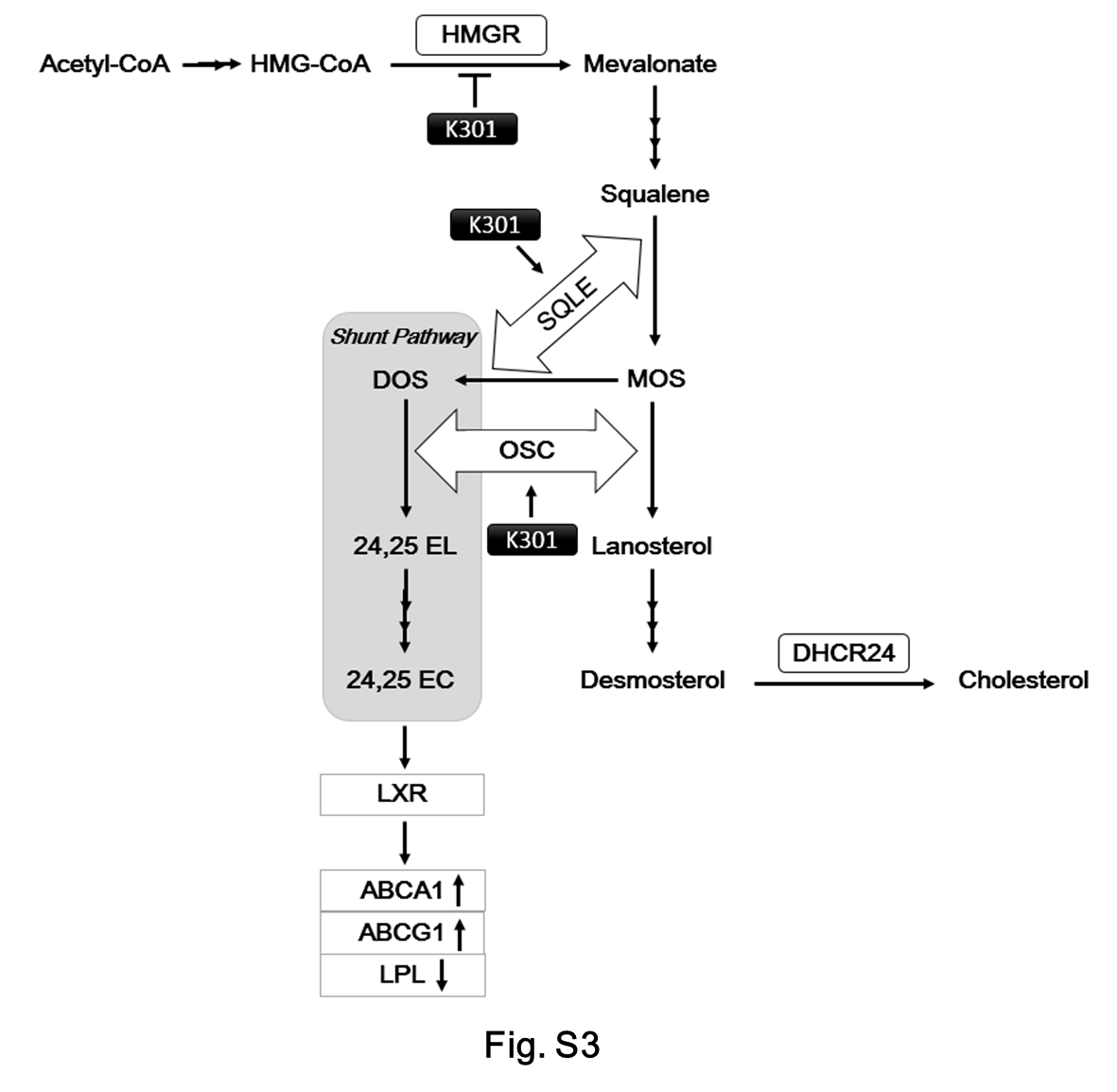

Supplement: S3 Fig — L. acidophilus K301 induces SQLE and OSC gene expression, which results in the induction of 24, 25-EC in the shunt pathway. 24, 25-EC activates LXR and LXR-related genes ABCA1 and ABCG1, while decreasing expression of LPS. L. acidophilus K301 inhibits cholesterol biosynthesis by degrading HMGR. (TIF) [file pone.0154302.s003.TIF]
