# Supplementary material for: Lactobacillus acidophilus K301 Inhibits Atherogenesis via Induction of 24 (S), 25-Epoxycholesterol-Mediated ABCA1 and ABCG1 Production and Cholesterol Efflux in Macrophages
Source: PLoS One. 2016 Apr 27;11(4):e0154302. doi: 10.1371/journal.pone.0154302 (PMC4847857; doi:10.1371/journal.pone.0154302)
Supplement: S1 Table — (DOCX) [file pone.0154302.s005.docx]

S1 Table

| **Product** | **Forward primer** | **Reverse primer** | **Size**  **(bp)** |
| --- | --- | --- | --- |
| ABCA1 | tgtccagtccagtaatggt | aagcgagatatggtccggat | 112 |
| ABCG1 | tgcaatcttgtgccatattt | ccagccgactgttctgatca | 108 |
| ApoE | ggtcgcttttgggatt | ttcaactccttcatggtc | 125 |
| ABCA1 promoter | aatgcagctaagttggaggtctggagtggctac | ccaaagcttgtcactggagagcctcttacctgt | 1,158 |
| CCR2 | taccaacgagagcggtgaag | gtagagcggaggcaggagtt | 117 |
| CD11b | ctgagcactgtggggagcta | cggcagcataacccaagtaa | 138 |
| CD36 | gcctctccagttgaaaaccc | tcccttctttgcatttgctg | 112 |
| LPL | gaagactcgttctcagatgc | cagagtgaatgggatgttct | 150 |
| SREBP-1c | ctgaactgtgtgacccagcc | ccatgctggaactgatggag | 159 |
| LXR-α | ggatcctatgtccttgtggctg | aagctttcattcgtgcacatcc | 1,488 |
| ß-actin | tggcacccagcacaatgaa | ctaagtcatagtccgcctagaagca | 196 |
| GAPDH | aggaggcattgctgatgatc | gtcttcaccaccatggagaa | 105 |
