# Supplementary material for: Lactobacillus acidophilus K301 Inhibits Atherogenesis via Induction of 24 (S), 25-Epoxycholesterol-Mediated ABCA1 and ABCG1 Production and Cholesterol Efflux in Macrophages
Source: PLoS One. 2016 Apr 27;11(4):e0154302. doi: 10.1371/journal.pone.0154302 (PMC4847857; doi:10.1371/journal.pone.0154302)
Supplement: S2 Table — (DOCX) [file pone.0154302.s006.docx]

S2 Table

| **Abbreviation** | **Lactic acid bacteria** |
| --- | --- |
| KCTC3164 | *Lactobacillus acidophilus* KCTC3164 |
| La14 | *Lactobacillus acidophilus* La14 |
| K301 | *Lactobacillus acidophilus* K301 |
| D3 | *Lactobacillus plantarum* D3 |
| D4 | *Lactobacillus rhamnosus* D4 |
| MK09 | *Bifidobacterium infantis* MK09 |
| BL720 | *Bifidobacterium longum* BL720 |
| B3 | *Lactobacillus paracasei* B3 |
| B4 | *Lactobacillus paracasei* B4 |
| LC107 | *Lactobacillus casei* LC107 |
